# Association between the use of a stylet in endotracheal intubation and postoperative arytenoid dislocation: a case-control study

**DOI:** 10.1186/s12871-018-0521-9

**Published:** 2018-05-31

**Authors:** Lingeer Wu, Le Shen, Yuelun Zhang, Xiuhua Zhang, Yuguang Huang

**Affiliations:** 1 Department of Anesthesiology, Peking Union Medical College Hospital, Chinese Academy of Medical Sciences and Peking Union Medical College, Beijing, China; 2 Central Research Laboratory, Peking Union Medical College Hospital, Chinese Academy of Medical Sciences and Peking Union Medical College, Beijing, China

**Keywords:** Arytenoid dislocation, Intubation stylet, Duration of operation, Case-control study, Postoperative complication

## Abstract

**Backgrounds:**

Arytenoid dislocation (AD) is a rare but severe complication after general anesthesia with endotracheal intubation. We conducted a case-control study at Peking Union Medical College Hospital to identify risk factors associated with AD, including the use of an intubation stylet.

**Methods:**

Patients who experienced AD were matched 1:3 with controls based on gender, age and type of surgery. Multiple conditional logistic regression was performed to determine associations between potential risk factors and AD.

**Results:**

Twenty-six AD cases were retrospectively identified from 2004 through 2016. On average, arytenoid dislocation occurred in 2 cases per year, with an incidence of 0.904/100,000 (approximately 0.01%). The 26 patients who experienced AD and 78 matched control patients were enrolled in this study. All enrolled patients underwent endotracheal intubation, and a stylet was used for intubation for 38.5% (10/26) of the AD patients and 64.1% (50/78) of the controls (OR = 0.23, 0.07–0.74). A higher incidence of AD was significantly associated with longer duration of operation (OR = 1.74, 1.23–2.47).

**Conclusions:**

The use of an intubation stylet for endotracheal intubation appears to protect against AD. Prolonged operation time increases the risk of AD. These factors should be considered when assessing the risks of AD associated with endotracheal intubation and in efforts to avoid this complication.

## Background

Arytenoid dislocation (AD) is a rare postoperative complication after general anesthesia with tracheal intubation [1]. The incidence of AD after general anesthesia has been reported to be approximately 0.01–0.1% [2]. The cause of AD may be inadvertent trauma to the cricoarytenoid joint from the insertion of airway tools into the larynx [3–5]. To our knowledge, no systematic investigations of AD associated with tracheal intubation have been reported. Here, we report a hospital-based, case-control study to identify risk factors for surgery-associated AD.

## Methods

This investigation was a retrospective hospital-based, case-control study approved by the Peking Union Medical College (PUMC) Hospital Institutional Review Board. The study protocol was designated S-K260 and was approved on May 11th, 2017. All the data were collected from the adverse-event system in Department of Anesthesiology. No written informed consent was obtained from the participants. A verbal consent to participation in this study of each participant was obtained through a phone call during follow-up, as all the participants had been discharged when we collected the data.

### Patients

From January 2004 to December 2016, 26 cases involving postoperative AD were reported in the adverse-event system. Patients were matched 1:3 (case:control without AD) based on gender, age and type of surgery. We also recorded the following information for each patient: body mass index (BMI), surgical history, pre-existing laryngeal disease, comorbidities, smoking and alcohol history, American Society of Anesthesiologists physical status classification, number of intubation attempts, Cormack Lehane grading during intubation, intubating tools, size of the endotracheal tube, duration of operation, postoperative admission to the intensive care unit, whether a stylet was used to assist with intubation, and preoperative baseline laboratory data.

### Diagnosis of arytenoid dislocation

All the patients with AD appear some similar symptoms within one week after the operations, such as, hoarseness, choking when drinking water, and cough. Then electronic fibro-laryngoscopy would be performed by attending otolaryngologists to make the final diagnostic reports.

### Statistical analysis

Categorical variables were expressed as frequencies and percentages, which were compared using chi-squared tests. Continuous variables were summarized as means ± SD and compared using Student’s unpaired t-test. We use Bonferroni correction on the top of simple multiple t-tests to adjust the statistical significant level. Multiple conditional logistic regression was performed to determine associations between potential risk factors and AD. Statistical analyses were conducted using SPSS 19.0 (SPSS Inc., Chicago, IL, USA).

## Results

A total of 287,767 cases involving surgery under general anesthesia were recorded at our hospital from 2004 to 2016, with an average of 22,136 such cases per year (Table 1). Twenty-six cases involving AD, an average of 2 such cases per year, were recorded; thus, the incidence of this complication was approximately 0.01% (0.904/100,000).

**Table 1:** Incidence of AD at the PUMCH from 2004 through 2016

Year	Total Cases of General Anesthesia	Total Cases of AD	Incidence of AD^1^ (per 100,000)
2004	13,160	1	0.760
2005	14,145	1	0.707
2006	14,666	0	0.000
2007	15,054	0	0.000
2008	15,731	0	0.000
2009	19,190	0	0.000
2010	20,941	2	0.955
2011	21,687	5	2.306
2012	24,960	5	2.003
2013	28,525	3	1.052
2014	31,857	3	0.942
2015	33,192	4	1.205
2016	34,659	2	0.577
Average Cases/Year	22,136	2	0.904
Total Cases	287,767	26	0.904

Table 2 lists basic information for the patients who experienced postoperative AD. These 26 patients included 14 males. Their ages ranged from 23 to 83 (54.0 ± 17.376) years. With respect to American Society of Anesthesiologists physical status classification, 8 patients, 15 patients, 2 patients, and 1 patient were categorized as class I, II, III, and IV, respectively. The types of surgery performed included various extensive procedures. In PUMC hospital, Macintosh direct laryngoscope is the primarily routine choice for intubation, and the endotracheal tube size is commonly ID 7.5 for male patients and ID 7.0 for female patients. All AD patients underwent tracheal intubation, and among them only one patient received 3 attempts of intubation with Shikani optical stylet. Three AD patients received a double-lumen bronchial tubes, the rest 23 AD patients received single-lumen endotracheal tubes, with none of them received a laryngeal mask airway. AD occurred on the left side in 15 patients and on the right side in 11 patients. No patients in the both group suffered pre-existing laryngeal disease or arthritis before the operation. All AD patients recovered or improved after reduction procedure.

**Table 2:** Basic Information of Patients with Intraoperative AD

Case ID	Gender	Age	ASA	Name of Surgery	AD side
A1	2	50–59	II	Combined liver resection	L
A2	2	40–49	I	Exploratory laparotomy, Phemister	R
A3	2	50–59	I	Radical operation for stomach carcinoma	R
A4	1	70–79	I	Bowel resection	L
A5	1	30–39	III	Vaginal stump bleeding in suture technique	L
A6	2	20–29	IV	Exploratory laparotomy	L
A7	2	20–29	I	Intracranial brain electrodes	L
A8	2	80–89	I	Open cholecystectomy	R
A9	1	60–69	II	Anterior approach decompression	L
A10	1	20–29	II	Exploratory laparotomy	R
A11	1	50–59	I	Hepatic lateral lobectomy	L
A12	1	70–79	II	Segmental hepatectomy	L
A13	2	40–49	I	Open thoracic exploration, aortic replacement	R
A14	1	70–79	II	Whipple	R
A15	1	60–69	III	CABG	L
A16	2	30–39	II	Aortic replacement	R
A17	2	60–69	II	Miles	L
A18	2	30–39	II	Aortic replacement	R
A19	1	70–79	II	Total knee arthroplasty	L
A20	2	60–69	II	Anterior approach decompression	R
A21	1	70–79	II	LVATS	L
A22	2	40–49	I	Whipple	L
A23	2	60–69	II	LVATS	L
A24	2	50–59	II	Radical operation for cardia carcinoma	R
A25	1	60–69	II	Retroperitoneal neoplasm resection	L
A26	2	40–49	II	Exploratory laparotomy	L

Table 3 presents comparisons between the cases and controls for numerous variables. The following differences were statistically significant. Relative to the controls, the AD patients had longer durations of operation and longer postoperative hospital stays, but less frequently underwent intubation involving the use of an intubation stylet. The duration of operation was 4.03 ± 1.67 h for the cases and 2.60 ± 1.52 h for the controls (*P* < 0.002). The two groups did not significantly differ with respect to age; gender; BMI; depth of intubation; frequency of postoperative admission to the intensive care unit; rates of hypertension, diabetes mellitus, smoking, or alcohol use; or results for several preoperative laboratory tests, including assessments of serum albumin concentration, alanine aminotransferase, prothrombin time, and activated partial thromboplastin time.

**Table 3:** Comparsion between Case and Control

Variables	Case (*n* = 26)	Control (*n* = 78)	*P* value
Age (mean ± std)	54 ± 17.37	54 ± 17.37	1.000
Male, N (%)	15 (57.7)	45 (57.7)	1.000
BMI (mean ± std)	23.50 ± 3.69	23.19 ± 3.35	0.6863
Length of Surgery (mean ± std)	4.03 ± 1.66	2.60 ± 1.51	0.0002
Depth of Intubation (mean ± std)	23.00 ± 1.85	22.60 ± 1.77	0.1366
Number of attempts > 1,n (%)	1 (3.85)	3 (3.85)	1.000
Cormack Lehane Grade I, n (%)	18 (69.2)	60 (76.9)	0.438
Postopertive Hospital Days (mean ± std)	27.64 ± 12.16	15.88 ± 9.71	< 0.0001
Past Surgical History, n (%)	8 (30.76)	23 (29.48)	1.000
Hypertension, n (%)	6 (23.07)	25 (32.05)	0.464
Diabetes, n (%)	4 (15.38)	12 (15.38)	1.000
Somking, n (%)	5 (19.23)	23 (29.48)	0.444
Alcohol Drinking, n (%)	5 (19.23)	14 (17.95)	1.000
Postoperative ICU Admit, n (%)	5 (19.23)	10 (12.82)	0.519
Stylet Assisted Intubation, n (%)	10 (38.46)	50 (64.10)	0.038
^a^ASA I-II, n (%)	23 (88.46)	63 (80.76)	0.551
Complete Cell Count			
RBC (10^12^/L), mean ± std	4.08 ± 0.739	4.38 ± 0.533	0.0268
WBC (10^9^/L), mean ± std	7.73 ± 2.98	6.503 ± 2.903	0.0127
Hemoglubin (g/L), mean ± std	123.81 ± 22.53	132.71 ± 19.74	0.0516
Platelet (10^9^/L), mean ± std	216.60 ± 82.60	221.62 ± 83.34	0.5845
Albumin (g/L), mean ± std	38.96 ± 5.72	40.73 ± 4.97	0.0911
ALT (U/L), mean ± std	75.23 ± 196.22	22.69 ± 31.29	0.2161
PT (s), mean ± std	12.81 ± 3.72	12.15 ± 1.361	0.9263
APTT (s), mean ± std	30.25 ± 11.45	28.46 ± 6.241	0.7073

a: American Society of Anesthesiologists

Table 4 illustrates results from a multiple logistic regression model used to quantify associations between potential risk factors and AD. Intubation with a stylet was associated with a significantly reduced risk of surgery-associated AD (OR = 0.23, 0.07–0.74, *P* < 0.01), and longer duration of operation was associated with a higher risk of AD (OR = 1.89, 1.31–2.74, P < 0.01). Frequency of postoperative admission to the intensive care unit, American Society of Anesthesiologists class III-IV status, BMI, and serum albumin concentration were not significant risk factors.

**Table 4:** Risk Assessment of Intraopeartive AD

Variables	Unadjusted OR	95% ^a^CI	*P* value
Use of Stylet	0.35	0.14–0.87	0.03
Duration of Operation	1.71	1.26–2.33	0.00
ASA	1.08	0.58–2.00	0.81
BMI	1.03	0.90–1.17	0.69
Tube Depth	1.10	0.88–1.38	0.39
Variables	adjusted OR	95% ^a^CI	*P* value
Use of Stylet	0.23	0.07–0.74	0.01
Duration of Operation	1.89	1.31–2.74	0.00
ASA	1.05	0.90–1.23	0.51
BMI	1.01	0.48–2.11	0.99
Tube Depth	1.23	0.94–1.62	0.14

a: confidence interval

## Discussion

AD is a complication that is known to occur in all surgical departments, and factors that may either cause or help to prevent AD should be considered. Our incidence rate of AD (approximately 0.01%) is consistent with rates reported by Szigeti et al. [1] and other researchers [2]. The primary finding of our study is that the use of an intubation stylet was a significant protective factor for AD. Although certain authors have reported possible disadvantages of stylet use, including an increased incidence of postoperative pharyngeal pain [6] and an increased frequency of sore throat [7] caused by removal of the stylet. As a teaching hospital, most intubation attempts were conducted by residents under 5 years, or even medical interns, unless the intubation is too difficult. So intubation with a stylet is strongly encouraged especially for those trainees. The reason might be that stylet could decrease violence of laryngeal scope during exposing the glottis. An incidental but noteworthy finding of our study is that all of the patients in the case group underwent intubation; none of these patients received laryngeal masks. Therefore, the use of a laryngeal mask could be an approach for avoiding the occurrence of postoperative AD. In addition, we found that the severity of a patient’s health status (specifically, being ASA class III-IV) and BMI were not related to the incidence of AD.

Another important finding of our study was that duration of operation was significantly longer for the AD patients than for the control patients. Logistic analysis showed that no stylet use and length of surgery were independent risk factors for AD. Other authors [8] have reported that prolonged duration of anesthesia was a significant risk factor for the occurrence of AD and suggested that pressure exerted by a tracheal tube is a cause of AD [8]. However, it is difficult to separate the effects of stylet use and length of surgery. Another of our observations was that hospital stay was significantly longer for the patients with AD than for the patients without AD; we suspect that this difference is related to complexity of the surgical procedure but not to stylet use.

We also found that compared with the control group, the case group had significantly lower preoperative peripheral red blood cell counts and significantly higher white blood cell counts; however, the blood counts of both groups were within the normal range, and logistic analysis showed that neither of these counts was an independent risk factor for AD. Therefore, the significance of these hematologic findings with respect to AD is unclear. Anemia or joint inflammation may lead to instability [9], but it is not known whether either of these phenomena is relevant to the cause of AD. The results of several other common laboratory tests were also not significantly associated with AD; thus, our study did not identify a preoperative laboratory test that could predict the occurrence of AD.

In our series, no cases of AD were recorded during 2006–2009. We suspect that the reason for this phenomenon is that the Department of Anesthesiology’s adverse-event system was improved after 2010, with better follow-up of anesthesiologists’ performance. So there should be some missing AD cases in the cohort, as is a major limitation for our research. So we need to improve our adverse event reporting system to achieve more cases in future.

This study has some other limitations. For example, this study was a retrospective case-control study and may therefore have incorporated selection bias. Age, gender and surgery department could not be analyzed in this investigation. Secondly, since postoperative AD is a rare complication, our case group contained only 26 patients. Large, multi-center prospective trials may be needed to conclusively establish whether the use of an intubation stylet protects against AD.

In conclusion, AD is a rare but severe complication after general anesthesia. Use of an intubation stylet appears to protect against AD, and longer duration of operation favors the occurrence of this complication. These factors should be considered when assessing the risks of AD associated with endotracheal intubation and in efforts to avoid this complication.

## Conclusion

The use of an intubation stylet for endotracheal intubation appears to protect against AD. Prolonged operation time increases the risk of AD. These factors should be considered when assessing the risks of AD associated with endotracheal intubation and in efforts to avoid this complication.
